# The Mediating Effect of Empathy between Mindfulness and Self-Leadership in Female University Students: A Cross-Sectional Study

**DOI:** 10.3390/ijerph192315623

**Published:** 2022-11-24

**Authors:** Mikyoung Lee, Mijung Jung

**Affiliations:** Department of Nursing, Kwangju Women’s University, Gwangju 62396, Republic of Korea

**Keywords:** mindfulness, empathy, self-leadership, university students

## Abstract

(1) Background: Acknowledging scant research on integrating mindfulness, empathy, and self-leadership among female university students, this study aimed to investigate the relationships among these three variables, as well as the mediating effect of empathy. (2) Methods: A cross-sectional design was employed with 127 female sophomores in a mindfulness-based liberal arts class at K Women’s University in South Korea. Participants completed a self-reported questionnaire measuring levels of mindfulness, empathy, and self-leadership. Data were analyzed with descriptive statistics and correlations between the variables using the SPSS 28 program. The Jamovi 2.2.5 program was used to analyze the mediating effect of empathy. (3) Results: The levels of mindfulness, empathy, and self-leadership were higher than in previous studies. Mindfulness was positively related to empathy (r = 0.407, *p* < 0.001) and self-leadership (r = 0.635, *p* < 0.001); empathy was also positively associated with self-leadership (r = 0.635, *p* < 0.001). Furthermore, empathy mediated the relationship between mindfulness and self-leadership (β = 0.187, *p* < 0.001). (4) Conclusions: The findings indicate that mindfulness is helpful in improving university students’ self-leadership by reinforcing their empathy, and that an integrated training program of mindfulness and empathy could produce positive effects on promoting self-leadership. The findings can be utilized as a basis for developing programs to improve mindfulness and empathy, eventually improving students’ self-leadership.

## 1. Introduction

Self-leadership is defined as a “process of influencing oneself” ([1], p. 5) that improves individuals’ effectivity and performance [2]. It is initiative-based leadership in which individuals set goals for themselves and motivate themselves to lead a successful life [3]. Given that self-leadership is strongly connected to motivational constructs such as self-regulation and self-efficacy [2], it is crucial for university students to develop self-leadership for a better adaptation to university life, academic performance, and psychological wellbeing [4]. Several studies have reported the benefits of self-leadership strategies. For example, self-leadership lowered stress and improved coping [5], and positive emotions were enhanced by applying self-leadership strategies [6]. In addition, some researchers found a positive relationship between self-leadership strategies and subjective wellbeing [7], and between self-leadership strategies and self-efficacy perceptions [8,9]. Since the university period is the time for students to prepare for participation in extended society, it is important to enhance their self-leadership skills for them to have a better future.

Mindfulness, described as intentional and nonjudgmental awareness paying attention to the present moment purposely [10], has been considered as one of the related factors in improving self-leadership [2]. Mindful attitudes are important for university students, since mindfulness plays a crucial role in not only reducing university-related stress and anxiety [11,12] but also fostering concentration, cognitive flexibility, working memory [13], academic self-efficacy [14], motivational achievement behaviors [15], and academic performance [16,17]. Mindfulness and self-leadership are related to each other via self-regulation processes, emphasizing self-focused observation to achieve the individual’s goals [2,18]. Self-leaders with high mindfulness possess high self-leadership skills and are well aware of present thoughts, emotions, and behaviors, while constantly attending to internal (e.g., thoughts and emotions) and external processes (e.g., social interactions) [19,20]. They behave more consciously and apply self-leadership strategies in a variety of situations more effectively [21]. Previous studies have reported a positive correlation between mindfulness and self-leadership [2,21,22]. 

Meanwhile, the literature has found that mindfulness was positively correlated with empathy among university students [23,24,25]. Considerable studies have also supported that mindfulness-based interventions improved university students’ empathy [26,27,28,29]. These findings conclude that it is possible for individuals to facilitate empathy through nonjudgmental self-awareness of their own emotions, which is one of the fundamental aspects of mindfulness [30,31,32,33]. 

Empathy is defined as affective and cognitive responses arising from another person’s emotional circumstances, which is equivalent to the other person’s emotions [34]. Empathy is a core life skill for healthy human relationships [35]. It also has been recognized as an important competence for university students who are starting to be engaged in new social connections [36]. Empathy enables students to understand others better by taking others’ perspectives, while being greatly aware of social situations [37]. To have empathy skills, understanding one’s own emotions should take precedence [38]. In other words, it is assumed that people with strong empathy skills already have the ability to recognize their own emotions and can further manage their emotions effectively. Thus, higher empathy could lead to higher self-leadership by being well aware of one’s own emotions and regulating those emotions in many situations. Previous findings reflect that empathy had a positive relationship with self-leadership [4,39] and was a strong predictor of self-leadership [40].

As such, prior studies on mindfulness, empathy, and self-leadership have mainly investigated the relationship between two variables out of the three and other additional variables such as communication [39], emotion regulation [41], or emotions [42]. Research that includes all three variables in one study to examine the relationships among them is still lacking. Therefore, this study aimed to investigate the relationships among mindfulness, empathy, and self-leadership in university students who were taking a mindfulness-based liberal arts class. In particular, this study explored the mediating effect of empathy in the association between mindfulness and self-leadership. The specific research questions were as follows:

1. What are the levels of mindfulness, empathy, and self-leadership in university students?
2. What is the relationship between mindfulness, empathy, and self-leadership in university students?
3. Does empathy mediate the relationship between mindfulness and self-leadership in university students?

Regarding research question 3, researchers have rarely investigated the mediating function of empathy between mindfulness and self-leadership among university students, although some found mediating effects of empathy in the link between nursing professional values and compassion satisfaction, as well as burnout [43]. Nevertheless, we chose empathy as a mediator in the relationship between mindfulness and self-leadership, following Baron and Kenny’s [44] suggestion that mediation could occur when there exist the following significant relationships: (1) between an independent and a dependent variable, (2) between an independent and a mediating variable, and (3) between a mediating and a dependent variable. For example, previous studies found significant positive relationships (1) between mindfulness and self-leadership [21], (2) between mindfulness and empathy [23,24], and (3) between empathy and self-leadership among university students [4,39]. Therefore, we explored the mediating role of empathy in the link between mindfulness and self-leadership.

## 2. Methods

### 2.1. Research Design

A cross-sectional design was employed to investigate the relationships among mindfulness, empathy, and self-leadership, as well as the mediating effect of empathy in female university students.

### 2.2. Participants and Procedure

The participants were 127 female sophomores who were taking a mindfulness-based liberal arts class at K Women’s University in G metropolitan city in South Korea. The average age of the participants was 21.22 years (SD = 0.84), ranging 20 to 26 years. The sample size was calculated using the G*power 3.1.9 software program. The sample size of 127 was suitable, reflecting the required number of 116 based on an effect size of 0.15, α probability of 0.05, power of 0.90, and five predictors in a path model using regression analysis. This met the minimum criteria of 104 for regression analysis [45]. Data were collected between 2 June and 10 July 2022. An online questionnaire was distributed to approximately 200 students in a mindfulness-based class. In total, 127 students completed the self-reported questionnaire. They read the research explanation and understood the research purpose before answering the questionnaire. Informed consent was obtained via online contact from all the participants who agreed to participate in the study. Then, they started answering the questionnaire on mindfulness, empathy, and self-leadership. It took about 10 min for the participants to complete the entire questionnaire. A small stationery item was provided to them as a reward for participation.

### 2.3. Ethical Considerations

This research was approved by the Institutional Review Board at K Women’s University in Korea (1041465-202206-HR-001-21). All processes were performed in accordance with ethical standards in studies involving human participants. Participants were assured that their responses would be used only for this research and would be kept confidential. They were also informed that they could stop participating in the study at any time without any penalty.

### 2.4. Measures

The instruments were composed of three measures to examine mindfulness, empathy, and self-leadership in university students. The total scores of all the measures were used for analysis, since the present research focused on investigating the associations between the main constructs of mindfulness, empathy, and self-leadership. Previous studies have also used the total score for measures, recognizing its effectiveness in keeping a parsimonious model in analysis [46]. The authors of this paper also wanted to start off by investigating a simple model first as a pioneering study on the relationships among mindfulness, empathy, and self-leadership. 

#### 2.4.1. Mindfulness

The participants’ level of mindfulness was measured using the Korean Cognitive and Affective Mindfulness Scale-Revised (CAMSR) [47]. The CAMSR was originally developed by Feldman et al. [48] and was validated in Korean by Cho [47]. This measure evaluates three subscales of mindfulness: attention (four items; e.g., “I am able to focus on the present moment”), awareness (four items; e.g., “It is easy for me to keep track of my thoughts and feelings”), and acceptance (two items; e.g., “I can accept things I cannot change”). Participants responded on a four-point Likert scale with the following choices: 1 (rarely), 2 (sometimes), 3 (often), or 4 (almost always). Cronbach’s alpha was 0.83 for the total scale, and the alphas of the subscales attention, awareness, and acceptance were 0.77, 0.68, and 0.63, respectively.

#### 2.4.2. Empathy

To measure the empathy level, the Interpersonal Reactivity Index (IRI), originally developed by Davis [49], was adopted. For the present participants, the Korean validated version of the IRI [50] was utilized. The IRI measures the aspects of cognitive empathy and affective empathy, with each empathy scale consisting of 15 items. One example item of the cognitive empathy scale is “I try to look at everybody’s side of a disagreement before I make a decision”); one example item of the affective empathy scale is “I often have tender, concerned feelings for people less fortunate than I”. Participants rated their answers on a five-point Likert scale ranging from 1 (strongly disagree) to 5 (strongly agree). Cronbach’s alpha was 0.89 for the total empathy scale. Regarding the two dimensions, Cronbach’s alpha coefficients were 0.81 for cognitive empathy and 0.84 for affective empathy, demonstrating good internal reliability.

#### 2.4.3. Self-Leadership

To assess the participants’ self-leadership, the Revised Self-Leadership Questionnaire (RSLQ) developed by Houghton and Neck [51] for university students was used. For the present sample, the Korean validated version of the RSLQ for university students [52] was applied. The RSLQ consists of the three dimensions of behavior-focused strategies (18 items; e.g., “I establish specific goals for my own performance”), constructive thought pattern strategies (12 items; e.g., “I visualize myself successfully performing a task before I do it”), and natural reward strategies (five items; e.g., “I focus my thinking on the pleasant rather than the unpleasant aspects of my school activities”). Participants answered the questionnaire on a five-point Likert scale ranging between 1 (strongly disagree) and 5 (strongly agree). Cronbach’s alpha was 0.94 for the total scale, and the alphas of the three dimensions, behavior-focused strategies, constructive thought pattern strategies, and natural reward strategies were 0.93, 0.94, and 0.92, respectively. 

### 2.5. Data Analyses

Data were analyzed with descriptive statistics (i.e., means, standard deviations, skewness, and kurtosis), and correlations between the main study variables were made using the SPSS 28.0 software program (IBM, Armonk, NY, USA). In addition, the Jamovi 2.2.5 program (http://www.jamovi.org/) was used to analyze the mediating effect of empathy in the association between mindfulness and self-leadership. 

## 3. Results

### 3.1. Levels of Mindfulness, Empathy, and Self-Leadership (Research Question 1)

Table 1 shows mean, standard deviation, skewness, and kurtosis values of the variables. The mean level of mindfulness was 2.82 (SD = 0.52) out of 4. The mean levels of empathy and self-leadership were 3.73 (SD = 0.47) and 3.71 (SD = 0.65) out of 5, respectively. The values of skewness and kurtosis of all the variables ranged between −2 and +2, demonstrating a normal distribution of the data [53].

### 3.2. Relationships among Mindfulness, Empathy, and Self-Leadership (Research Question 2)

Pearson correlation coefficients based on the measurements of mindfulness, empathy, and self-leadership are described in Table 2. The participants’ mindfulness was positively related to empathy (r = 0.407, *p* < 0.001) and self-leadership (r = 0.619, *p* < 0.001). The participants’ empathy was also positively related to self-leadership (r = 0.635, *p* < 0.001). All subcategories were positively associated with one another.

### 3.3. Mediating Effect of Empathy (Research Question 3)

**Figure 1:**
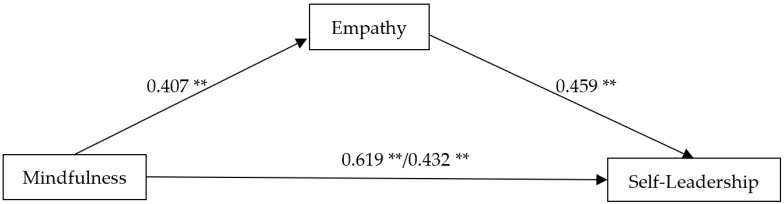
Mediation model of the effect of empathy between mindfulness and self-leadership; ** *p* < 0.001.

We tested mediation in a path model using the Jamovi 2.2.5 program. Table 3 and Figure 1 present the mediating effect of empathy in the relationship between mindfulness and self-leadership. When considering all variables in the model, mediation analysis showed that mindfulness had a significant positive influence on empathy (β = 0.407, 95% CI [0.224, 0.511], *p* < 0.001), and empathy also had a significant positive influence on self-leadership (β = 0.459, 95% CI [0.463, 0.816], *p* < 0.001). The direct effect of mindfulness on self-leadership was reduced after controlling for the effect of empathy (β = 0.432, 95% CI [0.384, 0.703], *p* < 0.001), compared with the total effect (β = 0.619, 95% CI [0.606, 0.951], *p* < 0.001). The indirect mediating effect through empathy was significant (β = 0.187, 95% CI [0.123, 0.347], *p* < 0.001), indicating that empathy partially mediates the relationship between mindfulness and self-leadership among university students. That is, the mediator’s participation augmented the level of self-leadership. Furthermore, the upper and lower limits of confidence intervals did not include zero in the mediation model, demonstrating a statistical significance.

## 4. Discussion

This study examined the levels of mindfulness, empathy, and self-leadership in female university students, as well as the relationships among these constructs. In particular, this study investigated the mediating effect of empathy in the link between mindfulness and self-leadership. Our main findings were that the three constructs of mindfulness, empathy, and self-leadership were positively related to one another, and that empathy mediated the association between mindfulness and self-leadership. More detailed discussions are provided below.

First of all, all three variables of mindfulness, empathy, and self-leadership in the present study showed a fairly higher score compared to those in existing studies that used the same measurements with Korean university students. For example, the mean for mindfulness in this study was 2.82 out of 4, higher than previous findings of 2.69 [16] and 2.26 [54]. The mean for empathy in this study was 3.73 out of 5, higher than earlier findings of 2.27 [39] and 3.48 [41]. In addition, the mean for self-leadership was also reported higher at 3.71 out of 5 in this study, compared to the previous scores of 3.10 [55] and 3.44 [56]. The present participants were sophomores who started taking a mindfulness-based liberal arts class from their first year at K Women’s University. It is mandatory for students at their university to take a mindfulness-based liberal arts course, consisting of one 1 h class per week for four semesters over a 2 year period. The constant mindfulness-based liberal arts class may have positively influenced the present participants’ mindfulness, empathy, and self-leadership, demonstrating relatively higher levels compared to prior findings with students who were not involved in a mindfulness-related class. Our findings are also supported by previous findings that mindfulness-based interventions improved the levels of mindfulness [57,58], empathy [31,57], and self-leadership [58] in participants.

The result of this study showed that mindfulness was positively related to empathy, indicating that students with mindful attitudes could enhance their empathy skills. This finding is consistent with numerous previous findings of a positive relationship between mindfulness and empathy [23,24,59,60,61]. This result corroborates previous findings that students with higher mindfulness tend to possess higher empathy. This result can also be explained by the fact that characteristics of compassion, kindness, and genuine behaviors with empathetic viewpoints are involved in current definitions of mindfulness [59,61]. Furthermore, the present finding of a positive correlation between mindfulness and empathy indicates that mindful awareness could foster empathy among university students. Focused self-awareness that is a key attribute of mindfulness enables individuals to become more aware of their own emotions; this can promote a better comprehension of emotional processes overall, ultimately leading to a better realization of present moment experiences [30,31,32,33]. Such an understanding helps individuals generate a greater capacity to consider others’ experiences including emotions, while being more empathetic.

This study found that mindfulness was also positively associated with self-leadership, in accordance with earlier findings [21,62,63]. Researchers have discussed that mindfulness and self-leadership are theoretically connected on the basis of self-regulation processes consisting of self-observation and goalsetting. For example, both constructs share certain similarities in that one of the core aspects of each is self-regulation of attention, and that each regards self-focused observation processes as important to reach one’s goals [2,18,20,21]. In addition, mindfulness emphasizes the nonjudgmental observation of the present moment in accomplishing tasks, and self-leadership is also concerned with enhancing personal effectiveness and outcomes [21]. Empirical findings further support the positive correlation between mindfulness and self-leadership. For instance, mindfulness and self-leadership improved self-efficacy and achievement behaviors, and reduced stress [64,65]. These discussions are reflected in this study’s finding of a positive association between mindfulness and self-leadership.

Furthermore, empathy was positively correlated with self-leadership. This suggests that students with a higher level of empathy are likely to possess a higher level of self-leadership. This result is consistent with previous findings of a positive correlation between empathy and self-leadership among university students [4,39]. There are some overlapping features between empathy and self-leadership. For example, self-leadership involves processes of self-motivation and self-regulation; in particular, self-regulation processes comprise self-observation and goalsetting [58]. Empathy also includes a self-observation factor; that is, when individuals are empathetic with others, they become very observant of the situations. Moreover, empathy is related to understanding of one’s inner experiences [66] and perceiving the world more accurately [67]. This suggests that individuals with higher empathy could have improved their self-understanding, their self-awareness, and the quality of their activities [68]. Attributes of empathy further involve accepting alternative perspectives about oneself, as well as understanding one’s situations [68]. These aspects of empathy could be connected to increasing one’s self-leadership, explaining the present positive relationship between empathy and self-leadership. In addition, for an effective application of natural reward strategies (one of three self-leadership dimensions; e.g., “I focus my thinking on the pleasant rather than the unpleasant aspects of my school activities”), individuals should have the ability to understand their own emotions first and regulate those emotions, which are pre-emptive conditions of robust empathy [38].

Lastly, mediation analysis supported that empathy mediated the relationship between mindfulness and self-leadership among the participating university students. This result confirms both the direct influence of mindfulness and the indirect influence of empathy on self-leadership. Specifically, mindfulness was positively associated with students’ self-leadership through increased empathy. This indicates that students with a higher level of mindfulness may possess better empathy skills, which in turn positively influences students’ improved self-leadership. It was found that empathy played the role of a partial mediator, suggesting that empathy explained only a certain portion of the link between mindfulness and self-leadership. Other factors might also exist to account for the detailed relationship between mindfulness and self-leadership. To the best of our knowledge, the present study is one of the pioneering investigations exploring the mediating role of empathy in the relationship between mindfulness and self-leadership. Future research should be conducted to examine the mechanism between these variables more thoroughly and comprehensively. Nevertheless, the present mediating effect of empathy confirms the benefit of empathy in the relationship between mindfulness and self-leadership among university students.

There are some limitations in this study that can be addressed in the future. One major limitation of this study is the sample size. Although the number of the present participants meets the minimum criteria for conducting regression analysis [45], we could have provided a better explanation of the model with a bigger sample size. It is necessary to conduct repetitive studies by expanding the number of the participants in the future. A second limitation is that a cross-sectional design was employed, and data were collected using self-reported measures. The possibility of participants providing biased responses cannot be excluded. Thus, the findings about causal relationships among mindfulness, empathy, and self-leadership, as well as the processes, should be cautiously interpreted. To compensate for this shortcoming, future studies could perform longitudinal research for a more thorough investigation of the causal relationships and exploration of trajectories. Future research could also incorporate qualitative data by conducting in-depth interviews or class observations and add more objective data from third parties. Another limitation is that this study included only female students who were taking a mindfulness-based liberal arts class at one women’s university in Korea. Therefore, the present findings are restricted to represent other populations including both male and female students, as well as students who did not take a mindfulness class. Future studies should involve a variety of samples from different universities in and outside Korea to replicate and expand the present study across genders and nationalities.

## 5. Conclusions

Acknowledging scant research on integrating the constructs of mindfulness, empathy, and self-leadership among university students despite their importance, the present study investigated the relationships among these variables. This study identified significantly positive associations among these variables, as well as the role of mindfulness and empathy as important factors influencing female university students’ self-leadership. Notably, this study is one of the rare studies confirming the mediating role of empathy in the relationship between mindfulness and self-leadership in university students. Our findings highlight that mindfulness would be helpful in improving students’ self-leadership by reinforcing their empathy.

The findings further imply that an integrated training program of mindfulness and empathy could have positive effects on promoting self-leadership in university students more effectively. These results can be utilized as evidence for developing and expanding programs to improve mindfulness and empathy in university students, eventually improving their self-leadership. Considering that self-leadership can be learned and reinforced [14,21,62], future research could be conducted to develop a program to improve self-leadership. When developing programs to improve self-leadership, researchers might include both mindfulness and empathy elements in the program based on the present study.

## Figures and Tables

**Table 1:** Mean, standard deviation, skewness, and kurtosis values of the variables.

Variables	Mean	SD	Min	Max	Skewness	Kurtosis
Mindfulness	2.82	0.52	1.30	3.90	0.10	−0.11
Empathy	3.73	0.47	2.07	4.87	−0.28	0.50
Self-Leadership	3.71	0.65	1.43	5.00	−0.04	0.62

**Table 2:** Correlations between the study variables.

Variables	1	1-1	1-2	1-3	2	2-1	2-2	3	3-1	3-2	3-3
1. Mindfulness	1										
1-1. Attention	0.883 **	1									
1-2. Awareness	0.861 **	0.631 **	1								
1-3. Acceptance	0.673 **	0.416 **	0.414 **	1							
2. Empathy	0.407 **	0.352 **	0.418 **	0.187 *	1						
2-1. Cognitive empathy	0.432 **	0.371 **	0.464 **	0.181 *	0.926 **	1					
2-2. Affective empathy	0.323 **	0.282 **	0.311 **	0.185 *	0.927 **	0.717 **	1				
3. Self-Leadership	0.619 **	0.554 **	0.617 **	0.279 **	0.635 **	0.633 **	0.543 **	1			
3-1. Behavior-focused strategies	0.582 **	0.530 **	0.596 **	0.224 *	0.575 **	0.562 **	0.503 **	0.947 **	1		
3-2. Constructive thought pattern strategies	0.538 **	0.474 **	0.517 **	0.286 **	0.663 **	0.661 **	0.568 **	0.924 **	0.778 **	1	
3-3. Natural reward strategies	0.594 **	0.521 **	0.589 **	0.291 **	0.456 **	0.487 **	0.358 **	0.838 **	0.695 **	0.762 **	1

** *p* < 0.001, * *p* < 0.05.

**Table 3:** Mediating effect of empathy between mindfulness and self-leadership.

				95% CI				
Type	Effect	Estimate	SE	Lower	Upper	β	z	*p*
Indirect	Mindfulness ⇒ empathy ⇒ self-leadership	0.235	0.057	0.123	0.347	0.187	4.10	<0.001
Component	Mindfulness ⇒ empathy	0.368	0.073	0.224	0.511	0.407	5.03	<0.001
	Empathy ⇒ self-leadership	0.639	0.090	0.463	0.816	0.459	7.10	<0.001
Direct	Mindfulness ⇒ self-leadership	0.543	0.081	0.384	0.703	0.432	6.69	<0.001
Total	Mindfulness ⇒ self-leadership	0.778	0.088	0.606	0.951	0.619	8.84	<0.001

## Data Availability

The data presented in this study are available upon request from the corresponding author.
